# The Trace Detection of Nitrite Ions Using Neutral Red Functionalized SH-β-Cyclodextrin @Au Nanoparticles

**DOI:** 10.3390/s18030681

**Published:** 2018-02-25

**Authors:** Xiaoyang Du, Xiaoxia Zhang, Chunlai Jiang, Weilu Zhang, Lizhu Yang

**Affiliations:** 1 College of Chemistry & Materials Engineering, Wenzhou University, Wenzhou, Zhejiang 325035, China; 15451283264@stu.wzu.edu.cn (X.D.); 16451283269@stu.wzu.edu.cn (X.Z.); 2 School of Pharmaceutical Sciences, Wenzhou Medical University, Wenzhou, Zhejiang 325035, China; 3 The Atmospheric Environment Department, Chinese Academy for Environmental Planning, Beijing 100012, China; jiangcl@caep.org.cn

**Keywords:** gold nanoparticles, cyclodextrin, neutral red, nitrite ions, fluorescence sensor, trace detection

## Abstract

A novel fluorescence sensor of NR-β-CD@AuNPs was prepared for the trace detection of nitrite in quantities as low as 4.25 × 10^−3^ μg∙mL^−1^ in an aqueous medium. The fluorescence was due to the host-guest inclusion complexes between neutral red (NR) molecules and gold nanoparticles (AuNPs), which were modified by per-6-mercapto-beta-cyclodextrins (SH-β-CDs) as both a reducing agent and a stabilizer under microwave radiation. The color of the NR-β-CD@AuNPs changed in the presence of nitrite ions. A sensor was applied to the determination of trace nitrites in environmental water samples with satisfactory results.

## 1. Introduction

Gold nanoparticles (AuNPs) have important applications in the fields of nanoscience and nanotechnology because of their unique optical, electronic, and catalytic properties [1]. First, the distance-dependent surface plasmon resonance (SPR) band of AuNPs makes them vital units for establishing assembly/disassembly modulated colorimetric sensors [2,3]. Second, the high specific surface areas of AuNPs result in their surfaces being modified with multiple ligands [4]. Meanwhile, AuNPs are an ideal energy acceptor in structured fluorescence resonance energy transfer systems (FRET) due to their high extinction coefficient [5,6,7]. Additionally, the major advantage of AuNPs-based sensors is that the molecular recognition can appear as a color change, which can be easily observed by the naked eye [8]. To date, AuNPs have been applied to the fabrication of assembly/disassembly modulated colorimetric sensors [9], as well as various types of optical [10,11] and electrochemical [12,13] sensors and biosensors [14]. Among them, the interactions of AuNPs with macrocycles such as cyclodextrins, calixarenes, and cucurbiturils [15,16,17] have received considerable attention for their special and potential properties, for example, the application of resveratrol-stabilized AuNPs in the anticancer field [18].

As a well-known molecular receptor, β-cyclodextrin (β-CD) can form host-guest inclusion complexes with a wide variety of organic, inorganic, and biologic guest molecules in their hydrophobic cavities [19,20]. In parallel, β-CD is water-soluble and environmentally friendly, and is useful in improving the dispersibility of the functional materials [21,22,23,24]. On the basis of host-guest interactions, these complexes have been well applied to self-assembly, drug/gene delivery, separation, and sensing applications [15,25]. Considering the unique topological structures that macrocyclic supramolecules possess, several novel3452 properties and corresponding new applications may be presented when β-CD is attached to the surfaces of AuNPs [26]. For example, β-CD-capped AuNPs assembled on ferrocene-functionalized indium tin oxide surfaces were applied to enhance the voltammetric analysis of ascorbic acid [27].

The concentration of NO_2_^−^ is one of the most important parameters in water quality [28]. The maximum allowable amount of nitrite in drinking water is 100 ng·mL^−1^, according to the regulation of the European Community [29]. The rapid detection of trace concentrations of NO_2_^−^ in water bodies is essential [30,31]. Many analytical methods for the trace detection of nitrite and nitrate have been reported, including colorimetric methods [32,33], fluorometric methods [34], and electrochemical methods [35,36]. However, these methods have limitations such as poor sensitivity, anti-interference, and the use of expensive experimental apparatus. The chemiluminescent methods have proven to be more sensitive and selective in the measurement of nitrite and nitrate [37,38,39,40,41,42]. Some of the typical methods are summarized in Table S1 (see Supplementary materials). Neutral red (NR) exists in two different prototropic forms in aqueous solutions, namely, the cationic/protonated (NRH^+^) and neutral (NR) forms, depending on the pH of the solution. NR is a type of dye containing a primary amine structure, which can interact with NO_2_^−^ and lead to fluorescence quenching. Meanwhile, it has been reported that hydroxyls in the cavities of β-CD form inclusion complexes with the nitrogen atoms on heterocyclic molecules of NR [43,44].

**Scheme 1:**
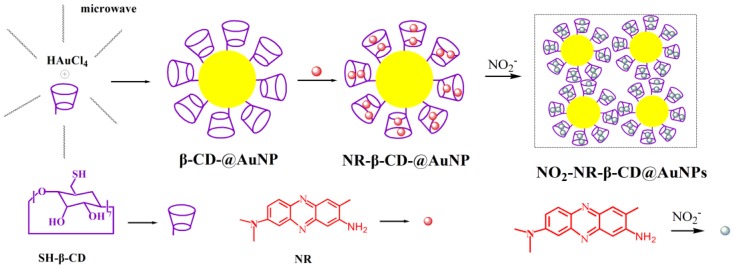
Schematic representation of the fabrication of the NR-β-CD@AuNP sensor and nitrite detection.

In this paper, a sensitive sensor is established for the trace detection of NO_2_^−^ in water because of the observation of a color change. Ultraviolet–visible spectroscopy (UV-Vis), transmission electron microscopy (TEM), and Fourier transform infrared spectroscopy (FT-IR) spectra are explored to understand the quenching interaction and corresponding binding forces. AuNPs modified by SH-β-CD were used as both the reducing agent and stabilizer in this method. Monodispersed β-CD@AuNPs with 10 nm diameters are synthesized in an eco-friendly way, which is different than previous approaches used for the fabrication of β-CD@AuNPs [45,46]. No harsh reagents are used in this method. NR-β-CD@AuNPs were synthesized by host-guest recognition between the β-CD@AuNPs and NR. The host was β-CD@AuNPs, and the guest was NR. The detection of nitrite ions was traced by the diazonium reaction of NO_2_^−^ and the primary amine of NR. The fabrication of the NR-β-CD@AuNP sensor and nitrite detection are shown in Scheme 1.

## 2. Materials and Methods

### 2.1. Reagents and Materials

Chloroauric acid trihydrate (HAuCl_4_·3H_2_O, 99.99%), sodium nitrite (NaNO_2_, 99.0%), hydrochloric acid (HCl, 36%), borax (Na_2_B_4_O_7_·10H_2_O, 99.0%), sodium bicarbonate (Na_2_CO_3_, 99.0%), neutral red (NR, 4% in water), sodium bicarbonate (NaHCO_3_, 99.0%), disodium hydrogen phosphate (Na_2_HPO_4_, 99.0%), sodium sulfate (Na_2_SO_4_, 99.0%), sodium chloride (NaCl, 99.0%), sodium fluoride (NaF, 99.0%), sodium dihydrogen phosphate (NaH_2_PO_4_, 99.0%), and sodium nitrate (NaNO_3_, 99.0%) were purchased from Aladdin Industrial Corporation (Shanghai, China). Per-6-mercapto-beta-cyclodextrin (SH-β-CD, 99.0%) was purchased from Shandong Binzhou Zhiyuan Bio-Technology Co., Ltd (Shandong, China). Other reagents were of analytical grade and directly used without further purification. All solutions were prepared using ultra-pure water (=18.20 MΩ·cm).

### 2.2. Apparatus

The morphology and the size of products were obtained from TEM, JEM-2100 (JEOL, Tokyo, Japan). The absorption spectra were obtained using a UV-2600 spectrophotometer (SHIMADZU, Tokyo, Japan). The fluorescence spectra were obtained using a FluoroMAX-4-TCSPC detector (HORIBA Jobin Yvon, Paris, France). The AuNPs were prepared with the microwave reactor Discover CEM (CEM, Matthews, NC, USA).

### 2.3. Preparation of the SH-β-CD Functionalized AuNPs (β-CD@AuNPs)

The β-CD@AuNPs were synthesized by the SH-β-CD reduction of HAuCl_4_. Briefly, 0.010 g HAuCl_4_·3H_2_O and 15.0 mg SH-β-CD were dissolved in 30.0 mL ultra-pure water using an ultrasonication for 5 min. The mixture was stirred for 3 min at 120°C under microwave radiation of 150 W. A suspension of the β-CD@AuNPs characterized by a wine-red color was finally obtained and stored at 4 °C. The reaction was different from previous approaches for the preparation of β-CD@AuNPs because no sodium borohydride was used.

### 2.4. Preparation of Fluorescence Dye-Incorporated SH-β-CD Functionalized Gold Nanoparticles (NR-β-CD@AuNPs)

In a typical experiment, 5 mL of a NaHCO_3_-borax buffer solution and 5 mL of NR (5 × 10^−6^ mol·L^−1^) were added into 5 mL of the β-CD@AuNP solution, and the solution was stirred in a dark environment at room temperature for 80 min. A solution of NR-β-CD@AuNPs was obtained, which became orange-red.

### 2.5. Detection of Nitrite Ions

NaNO_2_ (14.99 mg) was dissolved in ultra-pure water to prepare a 100.0 mg·L^−1^ standard solution, which was diluted to the desired concentrations for further use. A NaNO_2_ standard solution (2.1 mL) and a HCl (1.50 mg·L^−1^) solution (0.2 mL) were added sequentially into a 5-mL colorimetric tube, followed by the addition of 0.7 mL of the above-prepared NR-β-CD@AuNP solution. Fluorescence spectra were obtained after 5 min.

### 2.6 Detection of Nitrite Ions in Real Samples

The water samples were obtained from local ponds and Oujiang river (Wenzhou City, China). Then, the samples underwent filtration and centrifugal separation, after which the NR-β-CD@AuNPs were added to the samples, and then the fluorescence spectra were collected.

## 3. Results

### 3.1. Characterization of the β-CD@AuNPs

**Figure 1:**
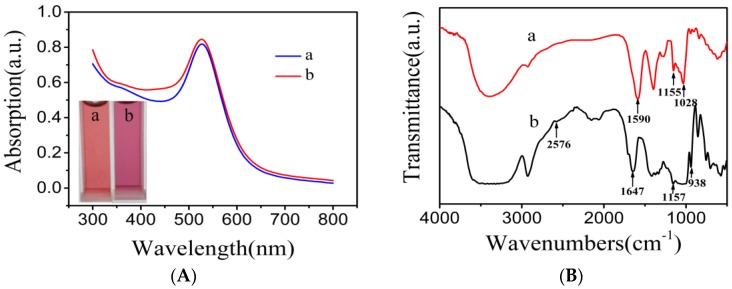
**(A)** UV-Vis spectra of the β-CD@AuNPs (a) and AuNPs (b); **(B)** FT-IR spectra of the β-CD@AuNPs (a) and SH-β-CD (b).

The UV-Vis spectra of the β-CD@AuNPs are shown in Figure 1A. An absorption band at 526 nm indicated the typical feature of the AuNPs and the localized surface plasmon resonance of the dispersed β-CD@AuNPs. The absorption peak was sharper than that prepared from HAuCl_4_ reduced by sodium citrate (see Supplementary materials, Figure S1A). The color also exhibited a slight variation that can be seen from the inner illustration of Figure 1A. The different colors of the AuNPs obtained using SH-β-CD and sodium citrate may be due to their different sizes and morphologies, as mentioned in Reference [47]. When the surfaces of the AuNPs were decorated with SH-β-CD molecules, they could be employed as scaffolds and energy acceptors for fluorescent sensing by host-guest interactions. The binding of SH-β-CD with AuNPs was verified by comparing the FT-IR spectra between the SH-β-CDs and β-CD@AuNPs, as shown in Figure 1B. The spectrum of SH-β-CD (b) had a band at 1647 cm^−1^ that corresponds to the stretching vibration peak of -C=O. The bands at 1157 and 938 cm^−1^ correspond to the stretching vibration peak of -C-O. The peak at 1590 cm^−1^ of the spectrum of β-CD@AuNPs (a) is the stretching vibration peak of -C=O. The two peaks at approximately 1155 and 1028 cm^−1^ correspond to the stretching vibration peak of -C-O. Moreover, the S-H stretching band at 2576 cm^−1^ of SH-β-CD (b) disappeared in the FT-IR spectrum of β-CD@AuNPs (a), which proved the formation of an Au-S bond, according to References [8] and [26].

**Figure 2:**
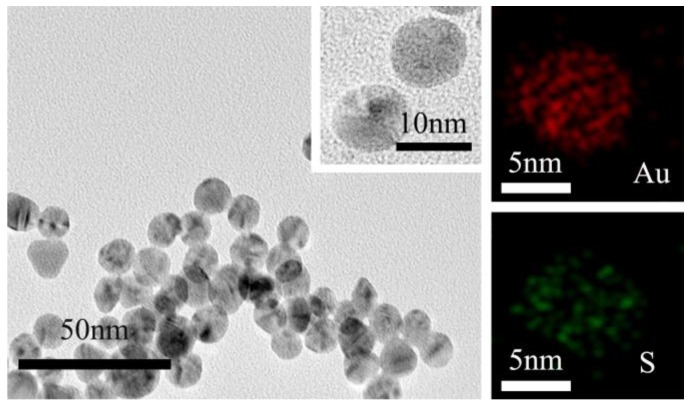
TEM images and energy dispersive spectrometry (EDS) element mappings of the β-CD@AuNPs; the red and green colors correspond to elemental Au and S, respectively.

To further confirm their nanostructure and atomic composition, the β-CD@AuNPs were analyzed by transmission electron microscopy (TEM), and the images are shown in Figure 2. The β-CD@AuNPs were nearly spherically shaped with an average size of 10 nm. Energy dispersive spectrometry (EDS) element mappings of β-CD@AuNPs are also shown in Figure 2 using different colors, in which the red and green areas correspond to elemental Au and S, respectively. 

The preparation of AuNPs from HAuCl_4_ reduced by sodium citrate was also tested in this paper. Additionally, the morphological characteristics of the TEM images are shown in Figure S1B (see Supplementary materials). Several of the nanoparticles were approximately 10 nm in size and some exhibited an irregular spherical shape.

### 3.2. Characterization of the NR-β-CD@AuNPs

**Figure 3:**
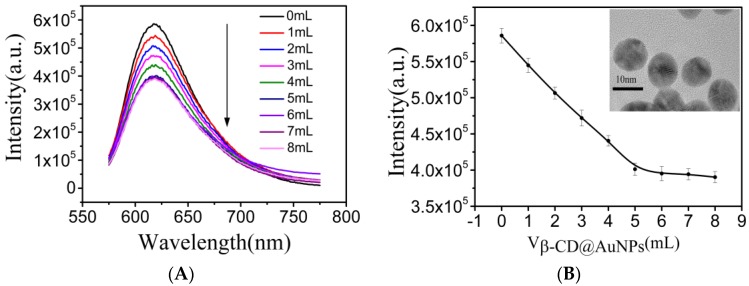
(**A**) Fluorescence spectra of the NR-β-CD@AuNPs when the volume of β-CD@AuNPs was 1, 2, 3, 4, 5, 6, 7, and 8 mL. (**B**) Plots of the NR-β-CD@AuNPs fluorescence intensities versus the volume of the β-CD@AuNPs with error bars. Inserted image: TEM image of the NR-β-CD@AuNPs.

To demonstrate the potential application of NR-β-CD@AuNPs during the trace detection of NO_2_^−^ in water, the host-guest recognition of β-CD@AuNPs and NR molecules was studied in this paper. Nitrogen heterocyclic molecules reacted with the hydroxyls of β-CD when the guest molecules of NR entered the cavities of the β-CD@AuNPs, used as the host molecules. The AuNPs were designed especially for their signal amplification in this paper. Therefore, a sensor of NR-β-CD@AuNPs exhibits a higher sensitivity than a sensor of both NR and NR-β-CD (see Supplementary materials, Figure S2A). The fluorescence of the NR-β-CD@AuNPs were gradually quenched with the addition of the host molecules of β-CD@AuNPs, and ultimately a stable quenching rate was attained when the volume of the β-CD@AuNPs was 5 mL. The fluorescence spectra are shown in Figure 3A. The relationship between fluorescence intensity and the volume of the β-CD@AuNPs solution is shown in Figure 3B. The fluorescence intensity of the NR-β-CD@AuNPs gradually decreased with an increase in β-CD@AuNPs. The fluorophores entered into the macrocyclic cavities of the β-CD@AuNPs for structure matching by host-guest interactions. As a consequence, the quenching efficiency achieved a constant value when the volume of the β-CD@AuNPs solution reached 5 mL. The average size of the NR-β-CD@AuNPs was 10 nm (see inserted TEM image in Figure 3B) and the dispersion of size was even and comparable to that of the β-CD@AuNPs (shown in Figure 2).

The energy of NR was transferred to the AuNPs through β-CD during the synthesis of NR-β-CD@AuNPs [47]. The quenching constant of K_sv_ was 1.68 × 10^4^ L·mol^−1^, which was calculated according to the Stern-Volumer equation [48]:

(1) $$F/F_{0}=1+K_{SV}\left[Q\right]$$

where *F*_0_ and *F* are the fluorescence intensities before and after the addition of β-CD@AuNPs, respectively; *K*_sv_ is the static quenching constant; and [Q] is the concentration of β-CD@AuNPs.

### 3.3. Effect of pH on the Fluorescence Property of the NR-β-CD@AuNPs

The prototropic equilibrium shifted from NRH^+^ to NR in the cavity of SH-β-CD with a change in the solution pH. NRH^+^ was the main form in an acidic aqueous solution with an absorption peak at 530 nm, and NR was the dominant form in a weakly alkaline media with an absorption peak at 450 nm. The fluorescence spectra of the NR-β-CD@AuNPs were changed with various pH values accordingly to the reaction between the NR-β-CD@AuNPs and NO_2_^−^, which could be clearly monitored by the fluorescence spectra. A diazonium group was formed by the selective reaction between NO_2_^−^ and the primary amine group of NR, which is unstable in weakly acidic and alkaline media, and rapidly converted to another stable form with nitrogen (N_2_) released [49]. On the other hand, diazonium salts easily react with surplus aromatic amine groups in NR with a deficiency of NO_2_^−^ during the diazotization reaction. As a result, the acid-base properties of the solution and the concentrations of NO_2_^−^ were the principal factors for the diazo coupling reaction.

**Figure 4:**
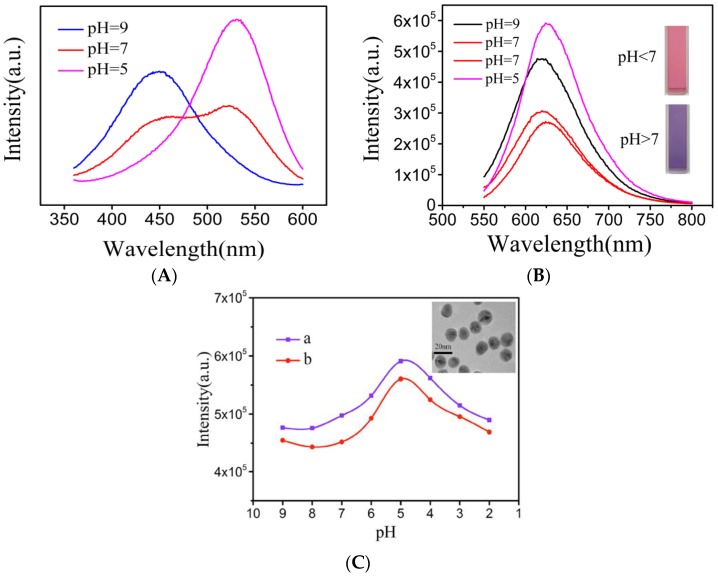
(**A**) Excitation spectra of the NR-β-CD@AuNPs at pH values of 9, 7, and 5; (**B**) Emission spectra of the NR-β-CD@AuNPs at pH values of 9, 7, and 5; (**C**) Fluorescence intensity of the NR-β-CD@AuNPs (a) and the NR-β-CD@AuNPs in the presence of NO_2_^−^ (100 μg·L^−1^) (b); Inserted image: TEM image of the NR-β-CD@AuNPs at a pH value of 5.

Experiments were carried out to explore the fluorescence properties of NR-β-CD@AuNPs at different pH values in the range of 2–9. The effect of pH on the excitation spectra of the NR-β-CD@AuNPs is shown in Figure 4A. The excitation peak appeared at 448 nm when the solution was weakly alkaline, which corresponds to the neutral form of NR. Another excitation peak appeared at 532 nm (pH = 7) due to the increasing amount of the protonated form of NRH^+^. Only the excitation peak at 532 nm remained when the aqueous solution was acidic. The corresponding emission peak (red line) shifted from 621 to 627 nm with an increase in the fluorescence intensity in Figure 4B. Because the ground-state pK_a_ value was 6.8 of NR in water, the critical point of the excitation peak at pH = 7 appeared. Changing from rose-red to purple, the colors of the NR-β-CD@AuNPs were different in weakly alkaline and acidic solutions, as shown in the inserted image of Figure 4B. The TEM image in Figure 4C shows the morphology of the NR-β-CD@AuNPs at pH 5, which is similar to that at pH 9. In the presence of NO_2_^−^, the fluorescence intensity of the solution clearly decreased compared to that of the NR-β-CD@AuNPs solution, as shown in Figure 4C. The NR-β-CD@AuNPs can detect trace amounts of NO_2_^−^ in acidic to weakly alkaline aqueous solutions. It was demonstrated that NR-β-CD@AuNPs have a broad detection range. The relatively wide detection range of NR-β-CD@AuNPs may have contributed to the structure of β-CD@AuNPs.

### 3.4. The Detection of NO_2_^−^ in an Aqueous Solution

**Figure 5:**
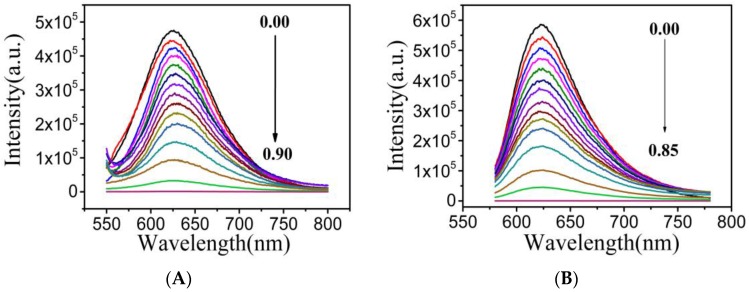
(**A**) Emission spectra of the NR-β-CD@AuNPs in the presence of different [NO_2_^−^] concentrations, including 0, 0.05, 0.10, 0.15, 0.20, 0.25, 0.30, 0.35, 0.40, 0.45, 0.50, 0.60, 0.70, 0.80, and 0.90 μg·mL^−1^ under a weakly alkaline medium; (**B**) Emission spectra of the NR-β-CD@AuNPs in the presence of different [NO_2_^−^] concentrations including 0, 0.05, 0.10, 0.15, 0.20, 0.25, 0.30, 0.35, 0.40, 0.45, 0.50, 0.60, 0.70, 0.80, and 0.85 μg·mL^−1^ under an acid medium.

To investigate the detection sensitivity of NR-β-CD@AuNPs to NO_2_^−^ in broad ranges, experiments were designed in both weakly alkaline and acidic media. When the solution was weakly alkaline, the fluorescence intensity of the NR-β-CD@AuNPs at 623 nm was clearly gradually quenched with an increasing concentration of NO_2_^−^ ([NO_2_^−^]), as shown in Figure 5A. A linear relationship (R^2^ = 0.998) was obtained between the fluorescence intensity and [NO_2_^−^] in the range of 0.0–0.9 μg·mL^−1^. When [NO_2_^−^] exceeded 0.9 μg·mL^−1^, the rate of fluorescence quenching reached 100%, and the color changed accordingly. The regression equation was F = 473893 − 539242C, where F represents the fluorescence intensity of the solution, and C represents [NO_2_^−^] (see Supplementary materials, Figure S3A). The detection limit was as low as 5.78 × 10^−3^ μg·mL^−1^, which was calculated as follows: the blank solution was measured 11 times, and its standard deviation was multiplied by 3 and divided by the slope of the linear relationship. The fluorescence quenching was static because the non-fluorescent diazonium groups were produced, and *K*_sv_ was 9.8 × 10^4^ L·mol^−1^, as calculated by Equation (1). The experiments under acidic conditions were performed in the same way as those performed under weakly alkaline conditions, apart from the employed pH values, and the results are shown in Figure 5B. The regression equation was F = 574156 − 673222C (see Supplementary materials, Figure S3B), and the detection limit was 4.25 × 10^−3^ μg·mL^−1^, which is better than that of the weakly alkaline conditions. The *K*_sv_ was 2.1 × 10^5^ L·mol^−1^, as determined by Equation (1).

**Figure 6:**
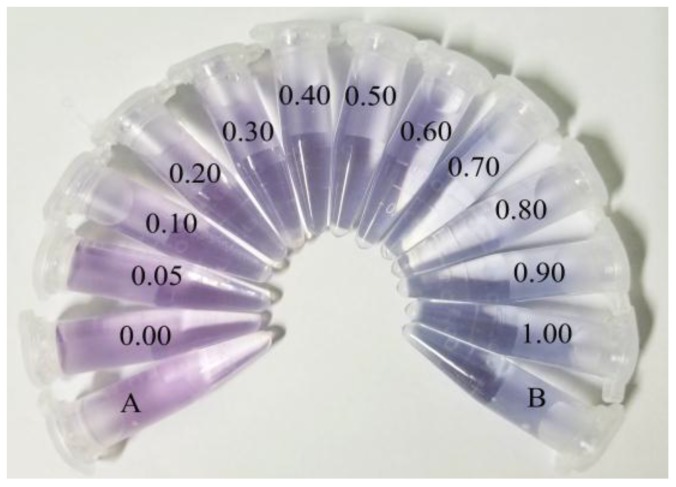
Photograph of the NR-β-CD@AuNPs in the presence of different [NO_2_^−^] concentrations including 0, 0.05, 0.10, 0.20, 0.30, 0.40, 0.50, 0.60, 0.70, 0.80, 0.90, and 1.00 μg·mL^−1^; A: 0 μg·mL^−1^ of [NO_2_^−^] and competing ions; and B: 1.00 μg·mL^−1^ of [NO_2_^−^] and competing ions.

As shown in Figure 6, the colorimetric response was recorded. It was obvious that the color changed from light purple to light blue, and could be observed by the naked eye, when [NO_2_^−^] was approximately 0.30 μg·mL^−1^.

Both NR and NR-β-CD could be used to detect NO_2_^−^ based on our results (see Supplementary materials, Figure S4), and the detection limit was 0.56 μg·mL^−1^ and 5.6 × 10^−2^ μg·mL^−1^, respectively. The solutions must be under a strongly acidic condition of pH 1 for higher detection limits. The NR-β-CD@AuNP sensor exhibited a good sensitivity of 5.78 × 10^−3^ μg·mL^−1^.

The diazonium group between NO_2_^−^ and the primary amine group of NR was more stable in an acid solution [49]. Compared with other sensors [50,51], this sensor displayed a wide detection range and good sensitivity. Some of the typical methods are summarized in Table S1 (see Supplementary materials).

**Figure 7:**
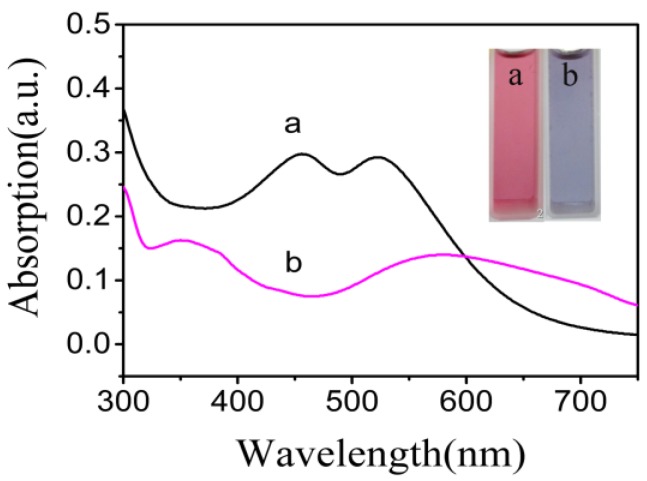
UV-Vis spectra of the NR-β-CD@AuNPs (a) and NO_2_-NR-β-CD@AuNPs (b).

After reacting with NO_2_^−^, the product of NO_2_-NR-β-CD@AuNPs was analyzed by UV-Vis spectra (Figure 7). The UV-Vis spectrum of the NR-β-CD@AuNPs (a) had two absorption bands at 520 nm and 450 nm, which correspond to the two states of NR. There were two new absorption bands appearing at 583 nm and 349 nm for the NO_2_-NR-β-CD@AuNPs. The color was also different when the NR-β-CD@AuNPs reacted with NO_2_^−^, as shown in Figure 7.

### 3.5. Selectivity

**Figure 8:**
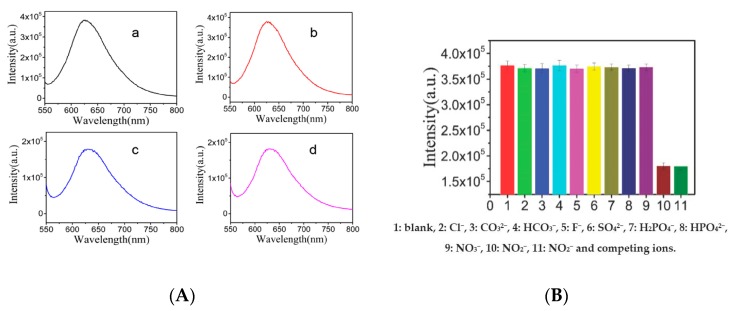
(**A**) Fluorescence spectra of the (a) NR-β-CD@AuNPs; (b) NR-β-CD@AuNPs and competing ions (Cl^−^, CO_3_^2−^, HCO_3_^−^, F^−^, SO_4_^2−^, H_2_PO_4_^−^, HPO_4_^2−^ and NO_3_^−^); (c) NR-β-CD@AuNPs and NO_2_^−^; and (d) NR-β-CD@AuNPs, NO_2_^−^ and competing ions. (**B**) Fluorescence intensity of the NR-β-CD@AuNPs and 1: blank, 2: Cl^−^, 3: CO_3_^2−^, 4: HCO_3_^−^, 5: F^−^, 6: SO_4_^2−^, 7: H_2_PO_4_^−^, 8: HPO_4_^2−^, 9: NO_3_^−^, 10: NO_2_^−^, 11: NO_2_^−^ and competing ions.

A 100-fold concentration of the other common ions were selected as competing ions in order to demonstrate the selectivity of this sensor, including Cl^−^, CO_3_^2−^, HCO_3_^−^, F^−^, SO_4_^2−^, H_2_PO_4_^−^, HPO_4_^2−^, and NO_3_^−^. Meanwhile, [NO_2_^−^] of 0.35 μg·mL^−1^ was measured as the control group. As shown in Figure 8, the fluorescence quenching only occurred at 623 nm in the presence of NO_2_^−^, even though it contained other ions. These results emphasized the high selectivity of the fluorescence sensor for the trace detection of NO_2_^−^.

### 3.6. Application of NO_2_^−^ Detection in Real Samples

The sensor was used to detect NO_2_^−^ in the waters of a local river or pond to determine the realistic efficiency of an NR−β−CD@AuNP sensor. Recovery experiments were carried out on samples by adding nitrite ion standards [52]. The data are listed in Table 1, and the method has a recovery of 98.6−102.5% with a relative standard deviation (RSD) of less than 3%. The result indicated that the sensor was reliable for detecting NO_2_^−^ in outdoor waters. It is confirmed that the NR−β−CD@AuNPs could be available for the detection of NO_2_^−^ in real samples.

## 4. Conclusions

A fluorescence sensor was fabricated by modifying β−CD@AuNPs with NR for the trace detection of NO_2_^−^. The optional condition for the sensor was an acidic aqueous solution, and the detection limit was as low as 4.25 × 10^−3^ μg·mL^−1^. This sensor can selectively recognize NO_2_^−^ through a visual color change from light purple or pink to light blue when the [NO_2_^−^] concentration is 0.30 μg·mL^−1^. When [NO_2_^−^] exceeded 0.9 μg·mL^−1^, the rate of fluorescence quenching reached 100%, and the color changed. The sensor was applied to the detection of NO_2_^−^ in local waters with a low detection limit, wide linear concentration range, good reproducibility, and anti−interference ability.

## Figures and Tables

**Table 1:** Results of the detection of nitrite ions in river (1) and pond (2) water (n = 6).

Samples	Content (NO_2_^−^, μg·mL^−1^)	Added (NO_2_^−^, μg·mL^−1^)	Found (NO_2_^−^, μg·mL^−1^)	Recovery (%)	RSD (%)
1	0.23±0.01	0.10	0.33±0.01	101.00±1.5	1.20±0.5
2	0.32±0.02	0.10	0.42±0.01	99.80±1.2	2.10±0.7

## Supplementary Materials

The following are available online at http://www.mdpi.com/1424-8220/18/3/681/s1. Figure S1: (A) UV-Vis spectrum of AuNPs; (B) TEM image of AuNPs, Figure S2: (A) Emission spectra of NR (a) NR-β-CD@AuNPs (b) and NR-β-CD(c); (B) TEM images of NO_2_-NR-β-CD., Figure S3: The regression equation in a weakly alkaline (A) and an acidic medium (B)., Figure S4: (A) Emission spectra of NR in the presence of different [NO_2_^−^], including 0, 0.10, 0.20, 0.30, 0.40, 0.50 and 0.60 μg∙mL^−1^. (B) Emission spectra of NR-β-CD in the presence of different [NO_2_^−^], including 0, 0.20, 0.40, 0.60, 0.80, 1.00, 1.20, 1.40, and 1.6 μg∙mL^−1^. Table S1: Comparison of the fabricated sensor with other reported sensors for nitrite ions.

## References

[1] Daniel M.C., Astruc D. (2004). Gold nanoparticles: assembly, supramolecular chemistry, quantum−size−related properties, and applications toward biology, catalysis, and nanotechnology. Chem. Rev..

[2] Saha K., Agasti S.S., Kim C., Li X., Rotello V.M. (2012). Gold nanoparticles in chemical and biological sensing. Chem. Rev..

[3] Kelly K.L., Coronado E., Zhao L.L., Schatz G.C. (2003). The optical properties of metal nanoparticles: The influence of size, shape, and dielectric environment. J. Phys. Chem. B..

[4] Rana S., Bajaj A., Mout R., Rotello V.M. (2012). Monolayer coated gold nanoparticles for delivery applications. Adv. Drug Delivery Rev..

[5] Yang X., Yang M., Pang B., Vara M., Xia Y. (2015). Gold nanomaterials at work in biomedicine. Chem. Rev..

[6] Sapsford K.E., Algar W.R., Berti L., Gemmill K.B., Casey B.J., Oh E., Stewart M.H., Medintz I.L. (2013). Functionalizing nanoparticles with biological molecules: developing chemistries that facilitate nanotechnology. Chem. Rev..

[7] Ray P.C., Fan Z., Crouch R.A., Sinha S.S., Pramanik A. (2014). Nanoscopic optical rulers beyond the FRET distance limit: fundamentals and applications. Chem. Soc. Rev..

[8] Liu C.W., Lian J.Y., Liu Q., Xu C.L., Li B.X. (2016). β−Cyclodextrin−modified silver nanoparticles as colorimetric probes for the direct visual enantioselective recognition of aromatic α−amino acids. Anal. Methods.

[9] Liu J., Lu Y. (2006). Preparation of aptamer−linked gold nanoparticle purple aggregates for colorimetric sensing of analytes. Nat. Protoc..

[10] Deeb C., Zhou X.A., Gerard D., Bouhelier A., Jain P.K., Plain J., Soppera O., Royer P., Bachelott R. (2011). Off−resonant optical excitation of gold nanorods: nanoscale imprint of polarization surface charge distribution. J. Phys. Chem. Lett..

[11] Stender A.S., Wang G.F., Sun W., Fang N. (2010). Influence of gold nanorod geometry on optical response. ACS Nano.

[12] Guo S.J., Wang E.K. (2007). Synthesis and electrochemical applications of gold nanoparticles. Anal. Chim. Acta.

[13] Serafin V., Eguilaz M., Agui L., Yanez−Sedeno P., Pingarron J.M. (2011). An electrochemical immunosensor for testosterone using gold nanoparticles–carbon nanotubes composite electrodes. Electroanalysis.

[14] Pingarron J.M., Yanez−Sedeno P., Gonzalez−Cortes A. (2008). Gold nanoparticle−based electrochemical biosensors. Electrochim. Acta.

[15] Dsouza R.N., Pischel U., Nau W.M. (2011). Fluorescent dyes and their supramolecular host/guest complexes with macrocycles in aqueous solution. Chem. Rev..

[16] Yang Y.W. (2011). Towards biocompatible nanovalves based on mesoporous silica nanoparticles. Med. Chem. Commun..

[17] Sun Y.L., Yang B.J., Zhang X.A., Yang Y.W. (2012). Cucurbit [7] uril pseudorotaxane−based photo responsive supramolecular nanovalve. Chem. Eur. J..

[18] Mohanty R.K., Thennarasu S., Mandal A.B. (2014). Resveratrol stabilized gold nanoparticles enable surface loading of doxorubicin and anticancer activity. Colloids Surf. B.

[19] Palanisamy S., Sakthinathan S., Chen S.M., Thirumalraj B., Wua T.H., Loub B.S., Liuc X.H. (2016). Preparation of β−cyclodextrin entrapped graphite composite for sensitive detection of dopamine. Carbohydr. Polym..

[20] Freeman R., Finder T., Bahshi L., Willner I. (2009). β−cyclodextrin−modified CdSe/ZnS quantum dots for sensing and chiroselective analysis. Nano Lett..

[21] Wayu M.B., Schwarzmann M.A., Gillespie S.D., Leopold M.C. (2017). Enzyme−free uric acid electrochemical sensors using β−cyclodextrin−modified carboxylic acid−functionalized carbon nanotubes. J. Mater. Sci..

[22] Du D., Wang M.H., Cai J., Zhang A.D. (2010). Sensitive acetylcholinesterase biosensor based on assembly of β−cyclodextrins onto multiwall carbon nanotubes for detection of organophosphates pesticide. Sens. Actuators B Chem..

[23] Pourjavadi A., Eskandari M., Hosseini S.H., Nazari M. (2017). Synthesis of water dispersible reduced graphene oxide via supramolecularcomplexation with modified β−cyclodextrin. Int. J. Polym. Mater. Polym. Biomater..

[24] Abbaspour A., Noori A. (2011). A cyclodextrin host–guest recognition approach to an electrochemical sensor for simultaneous quantification of serotonin and dopamine. Biosens. Bioelectron..

[25] Chen Y., Liu Y. (2015). Construction and function of cyclodextrin−based 1D supramolecular strands and their secondary assemblies. Adv. Mater..

[26] Guo Y.Q., Zhao Y.M., Lu D.T., Wu H.J., Fan M., Wei Y.L., Shuang S.M., Dong C. (2014). β−Cyclodextrin functionalized gold nanoparticles: Characterization and its analytical application for L−tyrosine. J. Inclusion Phenom. Macrocyclic Chem..

[27] Luo C.H., Zheng Z.H., Ding X.B., Peng Y.X. (2008). Supramolecular assembly of β−cyclodextrin−capped gold nanoparticles on ferrocene−functionalized ITO surface for enhanced voltammetric analysis of ascorbic acid. Electroanalysis.

[28] Kodamatania H., Yamazakib S., Saitoc K., Tomiyasua T., Komatsud Y. (2009). Selective determination method for measurement of nitrite and nitrate in water samples using high−performance liquid chromatography with post−column photochemical reaction and chemiluminescence detection. J. Chromatogr. A.

[29] Beamonte E., Bermudez J.D., Casino A. (2007). A statistical study of the quality of surface water intended for human consumption near Valencia (Spain). J. Environ. Manag..

[30] Ito K., Takayama Y., Makabe N., Mitsui R., Hirokawa T. (2005). Ion chromatography for determination of nitrite and nitrate in seawater using monolithic columns. J. Choromatogr. A.

[31] Zuo Y.G., Wang C.J., Van T. (2006). Simultaneous determination of nitrite and nitrate in dew, rain, snow and lake water samples by ion−pair high−performance liquid chromatography. Talanta.

[32] Zhang H., Qi S.D., Dong Y.L. (2014). A sensitive colorimetric method for the determination of nitrite in water supplies, meat and dairy products using ionic liquid−modified methyl red as a colour reagent. Food Chem..

[33] Aydın A., Ercan Ö., Taşcıoğlu S. (2005). A novel method for the spectrophotometric determination of nitrite in water. Talanta.

[34] Wang L.L., Li B., Zhang L.M., Zhang L.G., Zhao H.F. (2012). Fabrication and characterization of a fluorescent sensor based on Rh 6G−functionlized silica nanoparticles for nitrite ion detection. Sens. Actuators B Chem..

[35] Ojani R., Raoof J.B., Zarei E. (2006). Electrocatalytic reduction of nitrite using ferricyanide; Application for its simple and selective determination. Electrochim. Acta.

[36] Paixão T.R.L.C., Cardoso J.L., Bertotti M. (2007). Determination of nitrate in mineral water and sausage samples by using a renewable in situ copper modified electrode. Talanta.

[37] Lu C., Lin J.M., Huie C.W., Yamada M. (2004). Chemiluminescence study of carbonate and peroxynitrous acid and its application to the direct determination of nitrite based on solid surface enhancement. Anal. Chim. Acta.

[38] Pelletier M.M., Kleinbongard P., Ringwood L., Hito R., Hunter C.J., Schechter A.N., Gladwin M.T., Dejam A. (2006). The measurement of blood and plasma nitrite by chemiluminescence: Pitfalls and solutions. Free Radic. Biol. Med..

[39] He D.Y., Zhang Z.J., Huang Y., Hu Y.F. (2007). Chemiluminescence microflow injection analysis system on a chip for the determination of nitrite in food. Food Chem..

[40] Mikuška P., Večeřa Z. (2002). Chemiluminescent flow−injection analysis of nitrates in water using on−line ultraviolet photolysis. Anal. Chim. Acta.

[41] Mikuška P., Večeřa Z. (2003). Simultaneous determination of nitrite and nitrate in water by chemiluminescent flow−injection analysis. Anal. Chim. Acta.

[42] Zhang T., Fan H.L., Jin Q.H. (2010). Sensitive and selective detection of nitrite ion based on fluorescence super quenching of conjugated polyelectrolyte. Talanta.

[43] Singh M.K., Pal H., Koti A.S.R., Sapre A.V. (2004). Photophysical properties and rotational relaxation dynamics of neutral red bound to β−cyclodextrin. J. Phys. Chem. A.

[44] Mohanty J., Bhasikuttan A.C., Nau W.M., Pal H. (2006). Host−guest complexation of neutral red with macrocyclic host molecules: Contrasting pK_a_ shifts and binding affinities for cucurbit [7] uril and β−cyclodextrin. J. Phys. Chem. B.

[45] Li H., Chen D.X., Sun Y.L., Zheng Y.B., Tan L.L., Weiss P.S., Wang Y.Y. (2013). Viologen−mediated assembly of and sensing with carboxylatopillar [5] arene−modified gold nanoparticles. J. Am. Chem. Soc..

[46] Huang T., Meng F., Qi L. (2009). Facile synthesis and one−dimensional assembly of cyclodextrin−capped gold nanoparticles and their applications in catalysis and surface−enhanced Raman scattering. J. Phys. Chem. C.

[47] Zhao Y., Huang Y.C., Zhu H., Zhu Q.Q., Xia Y.S. (2016). Three−in−One: Sensing, Self−Assembly, and Cascade Catalysis of Cyclodextrin Modified Gold Nanoparticles. J. Am. Chem. Soc..

[48] Eftink M.R., Ghiron C.A. (1981). Anal Biochem, Fluorescence quenching studies with proteins. Anal. Biochem..

[49] Liu Y.L., Kang N., Ke X.B., Wang D., Ren L., Wang H.J. (2016). A fluorescent nanoprobe based on metal−enhanced fluorescence combined with Förster resonance energy transfer for the trace detection of nitrite ions. RSC Adv..

[50] Adarsh N., Shanmugasundaram M., Ramaiah D. (2013). Efficient reaction based colorimetric probe for sensitive detection, quantification, and on−site analysis of nitrite ions in natural water resources. Anal. Chem..

[51] Chen J.H., Pang S., He L.L., Nugen S.R. (2016). Highly sensitive and selective detection of nitrite ions using Fe_3_O_4_@SiO_2_/Au magnetic nanoparticles by surface−enhanced Raman spectroscopy. Biosens. Bioelectron..

[52] Huang X., Li Y.X., Chen Y.L., Wang L. (2008). Electrochemical determination of nitrite and iodate by use of gold nanoparticles/poly(3−methylthiophene) composites coated glassy carbon electrode. Sens. Actuators B Chem..

