# Supplementary material for: The Trace Detection of Nitrite Ions Using Neutral Red Functionalized SH-β-Cyclodextrin @Au Nanoparticles
Source: Sensors (Basel). 2018 Feb 25;18(3):681. doi: 10.3390/s18030681 (PMC5877208; doi:10.3390/s18030681)
Supplement: Supplementary file 1 [file sensors-18-00681-s001.pdf]

Supplementary Materials

The Trace Detection of Nitrite Ions Using Neutral Red Functionalized SH-β-Cyclodextrin @Au Nanoparticles

Figure S1. (**A**) UV-Vis spectrum of AuNPs; (**B**) TEM image of AuNPs.

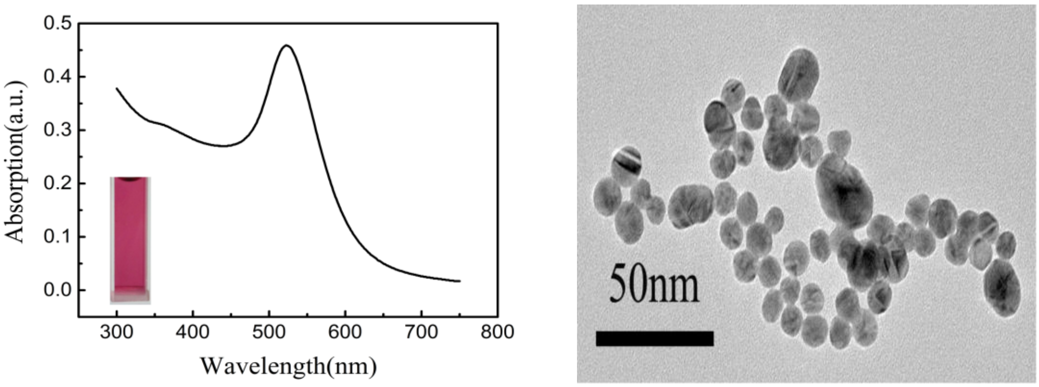

(A) **(B)**

AuNPs were prepared from $HAuCl_4$ by the reduction of sodium citrate. Briefly, 1 mL of $HAuCl_4$ solution with a mass percentage of 1% and 4 mL of sodium citrate with a 1% mass percent were added to 25 mL of ultra-pure water. The mixture was stirred for 10 minutes in a microwave reactor at 120°C with a microwave power of 150 W. β-CD@AuNPs was ultimately obtained as a purple liquid and was stored at 4°C.

The UV-Vis spectrum of AuNPs is shown in Figure S1A. An absorption band at 526 nm indicates a characteristic feature of AuNPs. The TEM image is

shown in Figure S1 B. Some of the particles were larger than 10 nm, and some had irregular spherical shapes.

Figure S2. (A) Emission spectra of NR (a) NR-β-CD@AuNPs (b) and NR-β-CD(c); (B) TEM images of NO_2-NR-β-CD.

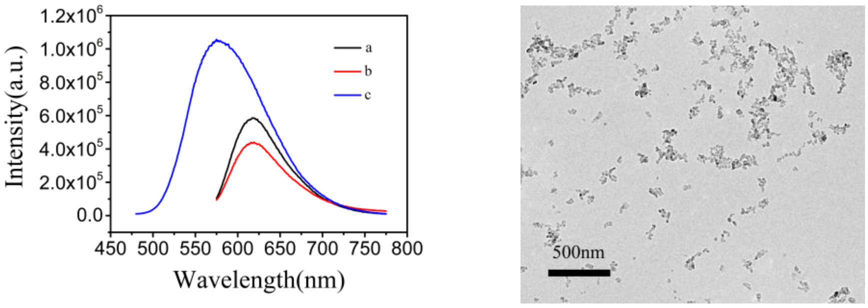

(A) (B)

Five milliliters of NR (5×10^{-6}mol·L^{-1}) was added to 10 mL of ultra-pure water, and the mixture was stirred in the dark at room temperature for 80 minutes to produce a solution of NR. In a typical case, 5 mL of $NaHCO_3$-borax buffer solution and 5 mL of NR (5×10^{-6}mol·L^{-1}) were added to 5 mL of ultra-pure water, and the mixture was stirred in the dark at room temperature for 80 minutes to produce a solution of NR-β-CD.

Then, 14.99 mg of $NaNO_2$ was dissolved in ultra-pure water to prepare 100.0 mg·L^{-1} of standard solution, which was diluted to the desired concentrations for further use. Next, 2.1 mL of $NaNO_2$ standard solution and 0.8 mL of HCl (1.50 mg·L^{-1}) solution were sequentially added into a 5-mL colorimetric tube, followed by an addition of 0.7 mL of NR-β-CD or NR solution as prepared above. The fluorescence spectra of NO_2-NR-β-CD or NO_2-NR were obtained after 5 minutes.

The emission spectra of NR (a), NR-β-CD@AuNPs (b) and NR-β-CD (c) are shown in Figure S2A. The emission peaks were 623 nm, 621 nm, and 523 nm, respectively. The TEM image of NO_2-NR-β-CD is shown in Figure S2B. The particles were small and formed an amorphous compound. In addition, these

compounds transform and disappear during transmission electron microscopy.

Figure S3. The regression equation in a weakly alkaline (A) and an acidic medium (B)

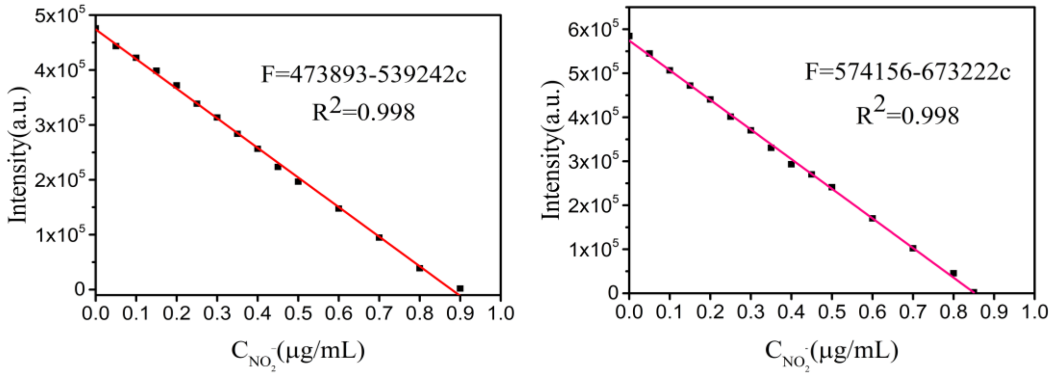

（A） （B）

To investigate the detection sensitivity of NR-β-CD@AuNPs to NO_2^- over broad ranges, experiments were designed in both weakly alkaline and acidic media. The resulting regression equations were F=473893-539242C (Figure S3 A) and F=574156-673222C (Figure S3 B), respectively, where F is the fluorescence intensity of the solution and C is [NO_2^-].

Figure S4. (**A**) Emission spectra of NR in the presence of different [NO_2^-], including 0, 0.10, 0.20, 0.30, 0.40, 0.50 and 0.60 μg·mL^{-1}. (**B**) Emission spectra of NR-β-CD in the presence of different [NO_2^-], including 0, 0.20, 0.40, 0.60, 0.80, 1.00, 1.20, 1.40, and 1.6 μg·mL^{-1}.

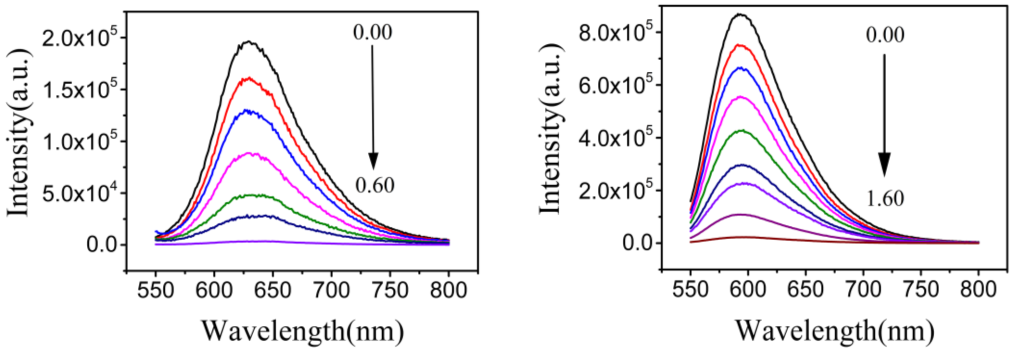

(A) **(B)**

It is clear that the fluorescence intensity of NR at 621 nm was gradually quenched as [NO_2^-] increased, as shown in Figure S4 (A). A linear relationship was obtained between the fluorescence intensity and [NO_2^-] over the range of

0.0-0.6 μg·mL^{-1}. The resulting regression equation was F=19358-30497C, and the detection limit was 0.56 μg·mL^{-1}. It is seen in Figure S4 (B) that the fluorescence intensity of NR-β-CD at 593 nm was gradually quenched as [NO_2^-] increased. The resulting regression equation was F=866342-500766C over the range of 0.0-1.6 μg·mL^{-1}, and the detection limit was 5.6×10^{-2} μg·mL^{-1}.

Table S1. Comparison of the fabricated sensor with other reported sensors for nitrite ions

Technique	Sample type	LOD (μg·mL^{-1})	Reference
colorimetric method	river water	7.5×10^{-3}	32
fluorometric method	water	5.52×10^{-2}	34
copper electrode	sausages	0.528	35
chemiluminescent method	tap and well water	4.9×10^{-3}	37
